# Clinical presentation and management of methanol poisoning outbreaks in Riyadh, Saudi Arabia: a retrospective analysis

**DOI:** 10.1186/s12873-024-00976-1

**Published:** 2024-04-16

**Authors:** Faisal Alhusain, Mohammed Alshalhoub, Moath Bin Homaid, Laila Carolina Abu Esba, Mohammad Alghafees, Mohammad Al Deeb

**Affiliations:** 1 Emergency Medicine Department, Ministry of the National Guard - Health Affairs, Riyadh, Saudi Arabia; 2 King Abdullah International Medical Research Center, Riyadh, Saudi Arabia; 3 Emergency Medicine Department, King Faisal Specialist Hospital and Research Center, Riyadh, Saudi Arabia; 4 Pharmaceutical Care Services, Ministry of the National Guard– Health Affairs, Riyadh, Saudi Arabia; 5 Surgery Department, Ministry of the National Guard - Health Affairs, Riyadh, Saudi Arabia

**Keywords:** Methanol toxicity, Methanol poisoning outbreak, Saudi Arabia

## Abstract

**Background:**

Acute methanol intoxication, whether unintentional or deliberate, necessitates prompt intervention to prevent severe morbidity and mortality. Homemade alcoholic beverages are a frequent source of such poisoning. This retrospective analysis examined two outbreaks of methanol intoxication in Saudi Arabia. It investigated the clinical presentation, implemented management strategies, and any lasting complications (sequelae) associated with these cases. The aim was to assess the potential impact of different treatment modalities and the timeliness of their initiation on patient outcomes.

**Methods:**

This was a retrospective case series of methanol poisoning cases which presented to the adult emergency department (ED) at King Abdulaziz Medical City (KAMC) in Riyadh, Saudi Arabia. There were two separate outbreaks in the city, the first one was from September 1 to September 10, 2020 and the second one was from May 14 to May 20, 2021. Electronic charts were reviewed, and data were extracted to previously prepared data extraction sheets.

**Result:**

From the 22 patients who arrived in the ED alive, the most common complaints were nausea or vomiting followed by altered level of consciousness. About 9% from the patient were hypotensive, 36% were tachycardic, 41% were tachypneic and 4% were having SpO2 < 94%. Brain CT was abnormal in 6 patients. Vision impairment was the most common sequalae of methanol poisoning (7 out of 12 patients who were assessed by ophthalmologist, 58%). When the patients were divided based on severity (mild, moderate, severe), nausea or vomiting and loss of consciousness were the most common complaints among the moderate group while loss of consciousness predominated in the severe group. Two patients presented with low blood pressure and were in the sever group. The severe group had a mean Glasgow Coma Scale (GCS) of 8. Most of the patients in the severity groups underwent the same management apart from those who died or deposited. Eight patients in the severe group had to be intubated.

**Conclusion:**

This study demonstrates the multifaceted clinical presentation of methanol poisoning, culminating in a 17.4% mortality rate. Notably, our findings emphasize the critical role of prompt diagnosis and swift initiation of combined fomepizole therapy and hemodialysis in mitigating mortality and minimizing the potential for chronic visual sequelae associated with methanol poisoning.

**Supplementary Information:**

The online version contains supplementary material available at 10.1186/s12873-024-00976-1.

## Introduction

Methanol is one of the poisonous alcohols frequently used as a solvent in automobiles, paint thinners and other industrial applications. Poisoning often arises from consumption of illicit or non-commercially produced alcoholic beverages, sometimes referred to as “moonshine.” These beverages inadvertently produce methanol during their synthesis [1]. Methanol poisoning, either accidental or intentional, is very harmful if not managed rapidly and may lead to significant morbidity and even mortality [2]. Methanol has a depressant effect on the central nervous system (CNS) when ingested or inhaled but the toxicity of methanol is attributed to its metabolite -formic acid- formed from the oxidation of methanol to formaldehyde and then to formic acid. Formic acid is toxic to the optic nerve, the CNS and the mitochondria and its concentration is directly related to the risk of morbidity and mortality [3]. Ingesting 50–100 milliliter of pure methanol can cause perpetual blindness and neurological deterioration resulting in death [4]. The clinical presentation of methanol poisoning varies according to the route of exposure, the amount ingested, and the elapsed time after ingestion. Early symptoms of methanol poisoning include nausea, vomiting, dizziness along with epigastric pain. Later -after a period of 12 to 48 h since ingestion- methanol poisoning can lead to neurologic dysfunction, blindness and even death. Metabolic acidosis with a high anion gap is the most prominent laboratory abnormality [5]. Various case studies have reported complications ranging from ischemia and necrosis to hypotension and coma [6, 7]. 

Managing methanol toxicity depends on the extent of exposure and requires close monitoring of laboratory parameters. Therapy with an antidote and/or extracorporeal treatment is the mainstay of treatment [8, 9]. The treatment approach is directed towards interrupting methanol breakdown to formic acid using a competitive alcohol dehydrogenase enzyme inhibitor, such as fomepizole or ethanol. In addition to directly eliminating the toxic metabolites through hemodialysis [4]. Administration of sodium bicarbonate is recommended to tackle metabolic acidosis and to reduce formic acid penetration into the CNS and optic nerve [3]. The use of folic acid is also recommended to accelerate the breakdown of formate [10]. Early administration of fomepizole has shown to reduce mortality and prevent the need for dialysis. In a multicenter prospective trial, fomepizole administration to 11 patients with methanol poisoning resulted in a fall in concentration of formic acid and an improvement in metabolic acidosis in all patients [11]. None of the 7 surviving patients that initially presented with visual abnormalities had any decrements in visual acuity at the end of the trial [11]. Dialysis is also required in severe cases to eliminate the toxic metabolite from the body, however a retrospective study reported a survival of 5 out of 15 patients (33.3%) who were treated with dialysis [9, 12]. 

The global significance of methanol toxicity has been underscored during the COVID-19 pandemic. Some regions witnessed methanol poisoning surges due to sanitizer consumption or misconceptions about alcohol’s protective effects against the virus. Notably, the outbreaks we describe, while coinciding with the pandemic, were linked to the illegal distribution of adulterated alcohol [12]. Given the profound health implications, including coma and death, early diagnosis and intervention are paramount. This study aimed to describe the clinical presentation, treatment strategies, and outcomes of patients from two distinct methanol poisoning outbreaks in Riyadh, Saudi Arabia, thereby filling existing knowledge gaps and underscoring the importance of timely public health interventions.

## Methods

### Study design

This study was a single center retrospective case series of methanol poisoning cases. It focused on patients that presented to the adult emergency department (ED) at King Abdulaziz Medical City (KAMC), a tertiary care academic hospital in Riyadh, Saudi Arabia. KAMC provides services to a rapidly growing patient population and houses 1,973 beds. The ED at KAMC offers care for national guard employees, their families, and critically ill or injured individuals. The study period encompassed two outbreaks between September 2020 and June 2021.

### Data collection

Data for this study were extracted from the electronic medical records at KAMC. The two documented outbreaks occurred from September 1 to September 10, 2020, and May 14 to May 20, 2021. Given the prohibition on the sale, purchase, and consumption of alcohol in Saudi Arabia [13]. As a result, some might resort to “illicit or non-commercially” alcohol produced illegally by local individuals in the country. For the scope of this study, 5 patients were considered from the first outbreak and 18 from the second. Diagnosis criteria depended on a positive methanol serum concentration exceeding 20 mg/dL. Details such as demographic information, symptoms upon arrival, initial vital signs, laboratory results, GCS, brain computed tomography (CT) findings, and treatment (encompassing fomepizole, sodium bicarbonate, dialysis, folate, and mechanical ventilation) were compiled. Additionally, assessments by ophthalmologist and/or neurologist were conducted for patients presenting with vision or neurological complaints.

### Data analysis

The gathered data were subjected to analysis using the Statistical Package for the Social Sciences (IBM SPSS Statistics for Windows, Version 22.0). Demographic data and baseline characteristics were summarized and presented as frequencies and proportions.

Based on the severity and clinical presentation, patients were categorized into three groups. A brief rationale for the grouping is: methanol poisoning’s severity can be gauged through clinical manifestations such as coma or seizures and laboratory indicators like blood pH levels. A lower pH often indicates acidosis, a common consequence of methanol poisoning. The severity groups were defined as: Mild: patients not in a coma, no seizures, and an initial pH > 7.2, Moderate: patients not in a coma, no seizures, but an initial pH ranging from 7.0 to 7.2 and Severe: patients in a coma, had seizures, or their initial pH was below 7.0. This classification helped in understanding the clinical implications of varying severities of methanol poisoning and guided subsequent interventions and prognosis evaluation.

## Results

### Patient demographics

A total of 23 patients presented to the ED over the two methanol toxicity outbreaks. The majority of patients were male (19/23, 83%) with a mean age of 29-years-old. Out of the 23 patients, one was pronounced dead on arrival, one died in the ED and the rest were discharged from the ED or were admitted for further management. Table 1 lists the initial presentation of the patients upon arrival to the ED. From the 22 patients who arrived alive to the ED, the most common symptoms were nausea or vomiting (17/22, 74%), altered level of consciousness (10/22, 44%), impaired vision (9/22, 39%) and abdominal pain (7/22, 30%). Only one patient presented asymptomatic with only a history of possible ingestion of methanol and positive methanol serum level. Two patients (9%) were hypotensive upon arrival, eight patients (36%) were initially tachycardic and nine patients were tachypneic (41%). All the patients (100%) had a normal initial temperature. Only eight patients from the total patients (35%) had brain CT and was abnormal in six patients, of which four showed brain edematous changes, and two had no brain perfusion and the rest were normal.

**Table 1:** Characteristics of patients

		N/mean	%
Gender	Male	19	83
	Female	4	17
Age (years)		29	10
Hospital Length of stay	< 24 h	4	17
	24–48 h	8	35
	> 48 h	11	48
Complaints	Nausea or vomiting	17	74
	Decrease level of consciousness	10	44
	Coma	2	9
	Blindness	1	4
	Impaired vision	9	39
	Dizziness	4	17
	Abdominal pain	7	30
	Seizure	3	13
	Asymptomatic	1	4
	Fever	1	4
	Arrest	1	4
Blood pressure	Normal	20	91
	Hypotension (SBP < 100)	2	9
Heart rate	Normal	14	64
	Tachycardic (> 100/min)	8	36
Respiratory rate	Normal	13	59
	Tachypnea (> 20/min)	9	41
SpO2	Normal	21	96
	< 94%	1	4
Mean Glasgow Coma Scale		12	4
Brain CT finding	Not done	15	65
	Normal	2	9
	Brain edematous changes	4	17
	No brain perfusion	2	9
Outcome	Impaired vision	7	30
	Cognitive impairment	5	22
	Brain death	3	13
Disposition from ER	Ward	9	39
	Intensive care unit	11	48
	Death	2	9
	Home	1	4
Final disposition	Home	17	74
	Death	4	17
	Leave against medical advice	2	9

### Patient outcomes

Table 2 characterizes those who presented to the ED with methanol poisoning according to severity. The groups were mild, moderate, and severe (3/23; 13%, 11/23; 48%, and 8/23; 35%, respectively). The mean age of the patients was 38 years old in the mild group, 27 years old in the moderate group and 28 years old in the severe group. All patients in the mild and moderate groups were males and only 50% of those in severe group were male. Nausea or vomiting and loss of consciousness were the most common complaints among the moderate group while loss of consciousness predominates in the severe group. Blood pressure readings were normal in the mild and moderate groups but was low (SBP < 100) in 25% of those in the severe group. Tachycardia (> 100 beats/min) among groups were as following: 33% in the mild group, 36% in the moderate group and 37% in the severe group. Tachypnea (> 20 breaths/min) was almost similar in the mild and moderate groups (33% and 27%, respectively). Tachypnea was almost doubled in the severe group (63%). Only 1 patient was having SpO2 < 94% and he was in the severe group. The mild and moderate groups showed an initial mean GCS of 14 and 15, respectively. The severe group, on the other hand, had a mean GCS of 8. VBG results showed a mean PH of 7.2 in the mild group, 7.1 in the moderate group and 6.8 in the severe group. HCO3 concentration had a mean of 15 mmol/l in the mild group, 9 mmol/l in the moderate group, and 14 mmol/l in the severe group. Mean methanol concentration was 136 mg/dl in the severe group, 177 mg/dl in the mild group and 113 mg/dl in the moderate group. White blood cells showed an upward trend among the groups: 9 cells per cubic millimeter in mild group, 14 cells per cubic millimeter in moderate group and 20 cells per cubic millimeter in severe group. In addition, creatinine was 139 µmol/L in the severe group while it was 78 µmol/L and 109 µmol/L in the mild and moderate groups, respectively. The mean anion gap (AGAP) and lactic acid were very high in the severe group (AGAP:25, Lactate:9 mmol/L). While the AGAP in the mild and moderate groups were 25 and 29 respectively and the Lactate were 1.84 mmom/L for mild group and 2.43 for the moderate group. Brain CT showed abnormal changes in certain patients in the severe group. Osmolality was 315 mOsm/kg in the mild group, 336 mOsm/kg in the moderate group and 362 mOsm/kg in the severe group.

**Table 2:** Participants according to severity groups

Variable	Category	pH > 7.2, no coma, no seizure		pH 7.0-7.2, no coma, no seizure		pH < 7.0, coma, or seizure	
		N/mean	%/SD	N/mean	%/SD	N/mean	%/SD
Gender	Male	3	16	11	58	4	21
	Female	0	0	0	0	4	100
Age (years)		38	28	27	3	28	5
Hospital Length of stay (days)		2	2	3	2	6	4
Hospital Length of stay	< 24 h	2	50	1	25	0	0
	24–48 h	0	0	5	63	3	38
	> 48 h	1	9	5	46	5	46
Complaints	Nausea or vomiting	2	12	10	59	5	29
	Decrease level of consciousness	0	0	4	40	6	60
	Coma	0	0	0	0	2	100
	Blindness	0	0	0	0	1	100
	Impaired vision	0	0	8	89	1	11
	Dizziness	0	0	4	100	0	0
	Abdominal pain	0	0	5	71	2	29
	Seizure	0	0	0	0	3	100
	Asymptomatic	1	100	0	0	0	0
	Fever	0	0	1	100	0	0
	Arrest	0	0	0	0	0	0
Blood pressure	Normal	3	100	11	100	6	75
	Hypotension	0	0	0	0	2	25
Heart rate	Normal	2	67	7	64	5	63
	Tachycardia	1	33	4	36	3	37
Respiratory rate	Normal	2	66	8	73	3	37
	Tachypnic	1	33	3	27	5	63
SpO2	Normal	3	100	11	100	7	88
	< 94%	0	0	0	0	1	12
Mean Glasgow Coma Scale		15	0	14	2	8	5
VBG	PH	7.233	0	7.123	0	6.778	0
	HCO3 (mmol/l)	15.5	3	9.1	2	13.9	26
	Pco2	36.8	6	27	3	35.3	18
Blood	WBC (cells/mm3)	8.96	3	14.34	6	19.72	6
	Methanol concentration (mg/dl)	117.44	69	112.82	46	136.79	74
	Ethanol concentration (mg/dl)	2	0	2	0	2	0
	Hemoglobin (gm/L)	231	109	159	49	159	15
	Platelet (^10^9/L)	249	50	316	100	394	60
	Potassium (mmol/L)	4.47	1	4.83	1	5.61	1
	Sodium (mmol/L)	134	3	135	2	136	5
	Creatinine (µmol/L)	78	11	109	19	139	54
	Glucose (mmol/L)	5.4	1	5.7	2	12.9	5
	BUN (mmol/L)	4.8	0	5.5	2	5.7	3
	ALT (U/L)	25	19	33	27	26	7
	AST (U/L)	25	7	28	6	32	8
	Amylase (U/L)	56	.	143	178	91	53
	AGAP (mmol/L)	25	9	29	6	37	7
	Lactic acid (mmol/L)	1.84	1	2.43	1	9.41	4
	Osmolality (mOsm/kg)	315	37	336	14	362	40
Brain CT finding	Not done	3	20	10	67	1	7
	Normal	0	0	1	50	1	50
	Brain edematous changes	0	0	0	0	4	100
	No brain perfusion	0	0	0	0	2	100
Fomepizole		3	14	11	50	8	36
Time to initiation of fomepizole (hours)	1	2	20	4	40	4	40
	2	0	0	2	67	1	33
	3	0	0	2	67	1	33
	4	0	0	1	100	0	0
	5	0	0	0	0	1	100
Dialysis		2	10	11	55	7	35
How many times of dialysis	1	1	8	8	67	3	25
	2	1	17	3	50	2	33
	3	0	0	0	0	2	100
Time to initiation of HD (hours)	1	0	0	0	0	1	100
	3	0	0	1	100	0	0
	4	1	14	4	57	2	29
	5	0	0	1	50	1	50
	7	0	0	0	0	2	100
	8	0	0	3	100	0	0
	10	0	0	1	50	1	50
	12	1	50	1	50	0	0
NaHCO3		3	13	11	48	8	35
Time to NaHCO3 initiation (hours)	1	2	18	3	27	6	55
	2	0	0	1	100	0	0
	3	0	0	3	75	1	25
	4	0	0	1	100	0	0
Folate	Yes	2	11	9	47	8	42
Intubation	Yes	0	0	2	20	8	80
Impaired vision	Yes	0	0	4	57	3	43
Cognitive impairment	Yes	0	0	0	0	5	100
Brain death	Yes	0	0	0	0	3	100
Ophthalmology evaluation	No available	1	9	4	36	5	46
	Impaired vision	0	0	4	57	3	43
	Normal	2	40	3	60	0	0
Disposition from ER	Ward	3	33	6	67	0	0
	Intensive care unit	0	0	4	36	7	64
	Death	0	0	0	0	1	50
	Home	0	0	1	100	0	0
Final disposition	Home	3	18	10	59	4	24
	Death	0	0	0	0	3	75
	Leave against medical advice	0	0	1	50	1	50

ED: Emergency department; HD: hemodialysis; NaHCO3: Sodium bicarbonate; VBG: Venous blood gas

The overall mortality rate was (4/23), 17.4%, three patients that died were in the severe group and one patient died up on arrival. Among those who were discharged from the hospital, vision impairment was the most common sequalae of methanol poisoning (7/12 who were assessed by ophthalmology, 58%), four patients (36%) in the moderate group and three patients (38%) in the severe group. Moreover, four patients (63%) in the severe group were diagnosed with brain death or edematous changes. Appendix 1 includes the full data for the patients.

### Patient managements

ED management included fomepizole, dialysis, sodium bicarbonate and folate. Almost 91% of the alive cases was started on hemodialysis (20/22). One of those who was not started on dialysis was in mild group and was asymptomatic and the other died in the ED before initiation of the dialysis. For mild group, one case was dialyzed for one time and the other had two sessions of dialysis while in the moderate group, eight cases had only one time of dialysis and three cases needed two sessions. Two cases in the severe group had three sessions of dialysis and two case needed three times of dialysis. The majority of the patients (11/20, 55%), were dialyzed within five hours or less from arrival to the ED. Twenty-two patients (22/22, 100%) in this study were started on fomepizole. All patients in the severe group had to be intubated (8/8, 100%) compared to two from the moderate group (2/11, 18%) and none from the mild group (0/3, 0%).

## Discussion

The multifaceted presentation of methanol poisoning poses a substantial diagnostic challenge, often presenting with a heterogenous constellation of symptoms across patients, potentially delaying suspicion and contributing to its significant morbidity and mortality [14]. However, prompt recognition and swift therapeutic intervention can dramatically mitigate the severity of sequelae [15]. Therefore, rapid source identification, coupled with proactive communication and heightened awareness amongst potentially exposed individuals, presents a significant opportunity for improved clinical outcomes. In our healthcare facility, timely diagnosis was achieved on the initial presentation itself, underscoring the critical role of early recognition in combating this potentially devastating toxicologic entity. The initial presenting complaint in this outbreak differed from previous reports. While nausea and vomiting were the most common symptoms observed, consistent with two prior outbreaks [14, 16], this contrasts with other studies where visual impairment was the dominant presentation [17–19]. The potential for ethanol co-ingestion, a less harmful alcohol, might explain this disparity, although further investigation is warranted. Upon emergency department presentation, a comprehensive laboratory evaluation including CBC, electrolytes, VBG, methanol, lactate, and osmolality was conducted based on clinical suspicion. Consistent with established literature [14, 16–18], the group with severe presentations exhibited the lowest mean serum pH, alongside the highest mean levels of methanol, potassium, lactate, WBCs, and osmolality. These findings underscore the importance of considering diverse presenting features in methanol poisoning, while highlighting the consistent laboratory profile associated with disease severity. All patients in the severe group, with the exception of the individual who demised before ICU admission, necessitated intensive care support. Notably, the severe group exhibited signs of nephrotoxicity, as evidenced by elevated mean creatinine (139 µmol/L) and blood urea nitrogen (BUN) levels (5.7 mmol/L). This observation aligns with the known nephrotoxic potential of methanol’s direct cytotoxic metabolite. While previous research suggests hypotension as a potential contributor to methanol-induced kidney injury [16], it is noteworthy that all patients within the hypotensive subgroup in this study also presented with renal impairment. These findings warrant further investigation to elucidate the precise mechanisms underlying and the potential interplay between hypotension and the direct cytotoxic effects of methanol metabolites in the pathogenesis of methanol-associated kidney injury. Upon suspicion of methanol poisoning based on a combination of clinical history, presentation, and metabolic acidosis, immediate therapeutic interventions were initiated as per the established local protocol. This aggressive management employed fomepizole to competitively inhibit methanol metabolism, sodium bicarbonate to rapidly rectify severe acidemia, folate for enhanced formic acid clearance, and hemodialysis for expeditious toxin removal. Notably, the observed mortality rate of 17.4% fell significantly below the average reported in other outbreaks (28-48%).^14,16^ This discrepancy can be primarily attributed to the swift diagnosis and prompt initiation of fomepizole and hemodialysis therapy, in contrast to delays or limited availability noted in previous reports. Notably, the majority of patients received fomepizole and dialysis within 3–5 h of emergency department arrival. While a slight delay in fomepizole administration for the initial case occurred, swift recognition of a potential influx of cases triggered a multi-faceted response. This led to operational collaboration and ensured timely fomepizole access for subsequent patients, prompting a comprehensive review of all antidote availability and distribution protocols, detailed elsewhere [20]. Several prior investigations have established a correlation between the severity of metabolic acidosis and mortality in methanol poisoning, aligning with our observations in this study. Patients who demised in the emergency department exhibited pH values below 7. Notably, four individuals within our cohort presented with similarly low pH (< 7), yet three experienced favorable outcomes and were discharged home, with one leaving against medical advice [14, 16–18]. This apparent discrepancy may be attributed to the prompt administration of fomepizole, the swift initiation of hemodialysis, and the number of dialysis sessions undergone. These findings suggest that an aggressive combined therapeutic approach, targeting both metabolic acidosis correction and toxin elimination, may mitigate the adverse prognostic implications associated with severe acidosis in methanol poisoning. Further research is warranted to elucidate the precise interplay between acidosis severity, early intervention, and ultimate prognosis in this complex clinical entity.

Early fomepizole administration can be crucial in preventing death and disability from methanol poisoning, as highlighted in a previous case series from our region with nine cases [21]. This is particularly important in Saudi Arabia, where alcohol consumption is prohibited due to religious and health reasons. However, there have been multiple outbreaks of methanol poisoning, especially among young people, during the COVID-19 pandemic. The pandemic likely played a role in these outbreaks by disrupting access to regulated alcoholic beverages, potentially leading to increased consumption of unregulated and often methanol-contaminated alternatives. For healthcare systems, this emphasizes the importance of having readily available stocks of essential antidotes like fomepizole and hemodialysis equipment, which can be lifesaving in such cases. Additionally, ongoing education for healthcare providers on the clinical management of toxic alcohol ingestions and the potential for outbreaks, particularly during public health crises, is crucial. By taking these steps, we can be better prepared to respond to future methanol poisoning outbreaks and improve patient outcomes.

## Conclusion

This study demonstrates the diverse clinical presentation of methanol poisoning, encompassing a spectrum of gastrointestinal, ophthalmic, and central nervous system manifestations. Notably, the observed low mortality and morbidity rate can be primarily attributed to the prompt diagnostic approach, swift initiation of fomepizole therapy, and rapid deployment of hemodialysis. These findings underscore the paramount importance of prioritizing early recognition and intervention in emergency departments during suspected methanol poisoning outbreaks. Establishing standardized protocols for expedited clinical assessment and laboratory testing, particularly in regions with a higher prevalence of unregulated alcohol consumption, holds crucial value in mitigating the potential morbidity and mortality associated with this toxicological entity.

### Electronic supplementary material

Below is the link to the electronic supplementary material.

Supplementary Material 1

## Data Availability

The datasets generated during the current study are available.
