# Supplementary material for: Clinical presentation and management of methanol poisoning outbreaks in Riyadh, Saudi Arabia: a retrospective analysis
Source: BMC Emerg Med. 2024 Apr 16;24:64. doi: 10.1186/s12873-024-00976-1 (PMC11020920; doi:10.1186/s12873-024-00976-1)
Supplement: Supplementary file 1 — Supplementary Material 1 [file 12873_2024_976_MOESM1_ESM.pdf]

Table S1: complete clinical data for all patients

#	Gender	Age	LOS (days)	Nausea/Vomiting	level of conscio	Coma	Blindness	impaired vision	Dizziness	Abdominal pain	Seizure	Asymptomatic	Fever	Arrest	SBP	DBP	HR	RR	SpO2	Temp	GCS	
17	Female	20 - 29	7	Yes	Yes	No	No	No	No	No	Yes	No	No	No	123	103	77	16	100	36.7	3	3
21	Male	20 - 29	4	No	No	Yes	No	No	No	No	Yes	No	No	No	160	100	102	122	100	36.5	6	3
20	Male	30 - 39	2	Yes	Yes	No	Yes	No	No	No	No	No	No	No	69	36	53	14	91	37	4	3
7	Male	20 - 29	11	Yes	Yes	No	No	Yes	No	Yes	No	No	No	No	145	87	129	24	94	36.9	13	3
12	Female	20 - 29	2	Yes	Yes	No	No	No	No	No	No	No	No	No	138	88	94	24	100	36.5	3	3
18	Male	30 - 39	12	No	No	Yes	No	No	No	No	No	No	No	No	90	52	95	32	95	38.2	8	3
13	Female	20 - 29	5	No	Yes	No	No	No	No	No	Yes	No	No	No	112	62	109	22	95	37	14	3
19	Female	20 - 29	2	Yes	Yes	No	No	No	No	Yes	No	No	No	No	119	75	98	20	100	36.7	15	3
6	Male	20 - 29	4	Yes	No	No	No	No	No	No	No	No	Yes	No	114	57	88	20	99	36.8	15	2
2	Male	30 - 39	2	Yes	No	No	No	Yes	No	Yes	No	No	No	No	110	58	66	20	99	36.2	15	2
22	Male	20 - 29	2	Yes	No	No	No	Yes	Yes	No	No	No	No	No	122	774	81	18	98	36.6	15	2
14	Male	30 - 39	4	Yes	Yes	No	No	No	No	No	No	No	No	No	154	141	147	90	99	36.6	10	2
23	Male	20 - 29	1	Yes	No	No	No	No	No	No	No	No	No	No	117	56	121	20	98	37	15	1
16	Male	20 - 29	0	No	No	No	No	No	No	No	No	No	No	Yes								
11	Male	20 - 29	1	No	No	No	No	No	No	No	No	Yes	No	No	133	82	70	21	99	36.6	15	1
10	Male	20 - 29	0	No	Yes	No	No	Yes	Yes	Yes	No	No	No	No	118	65	66	20	99	36.9	15	2
4	Male	20 - 29	2	Yes	Yes	No	No	Yes	No	Yes	No	No	No	No	149	99	105	19	99	36.4	15	2
5	Male	20 - 29	3	Yes	No	No	No	Yes	No	Yes	No	No	No	No	136	85	140	24	100	36.6	15	2
9	Male	20 - 29	3	Yes	No	No	No	Yes	No	Yes	No	No	No	No	138	86	79	20	97	26.8	15	2
3	Male	30 - 39	2	Yes	No	No	No	Yes	Yes	No	No	No	No	No	117	75	108	20	96	36.9	15	2
15	Male	20 - 29	2	Yes	No	No	No	Yes	Yes	No	No	No	No	No	138	89	91	26	98	36.9	15	2
1	Male	>69	4	Yes	No	No	No	No	No	No	No	No	No	No	114	100	65	20	98	36.9	15	1
8	Male	20 - 29	6	Yes	Yes	No	No	No	No	No	No	No	No	No	163	93	58	20	100	36.5	8	2

#	VBG; PH	VBG; HCO3	VBG; PCo2	WBC	Methanol Level	Ethanol level	Hemoglobin	Platelet	Potassium	Sodium	Creatinine	Co2	Glucose	BUN	ALT	AST	Amylase	AGAP	Lactic acid	Osmolality	Brain CT finding	Fomepizole
17	6.69	7.7	63.5	17.2	166.52	< 2	148	362	5.3	139	133	5	23.2	1.9	28	42	96	42	14.83	413	edema. No brain	Yes
21	6.8	5.3	35	27.7	214	< 2	181	434	5.4	140	200	5	10.6	5.9	29	27		46	11.28	390	edema. No brain	Yes
20	6.69	5.4	45.9	16.6	237.16	< 2	178	301	7	143	146	5	8.7	5.4	27	27		44	13.3		Brain edema with cerebellur	Yes
7	6.7	7.7	48	29.1	188.3	< 2	164	372	6.1	133	136	5	14	9.6				24	7.94	407	edematous cha	Yes
12	6.94	3.3	15.6	21.2	70	< 2	155	474	5.3	139	105	5	13.7	3.6	20	26		35	8.4	338	stemic hypodenc	Yes
18	6.69	4.9	23.3	13.7	85.4	< 2	158	341	5.7	126	232	5	14.2	9	39	44	162	36	9.42	330	hypotensity neu	Yes
13	7.08	3.4	11.8	16.7	41.1	< 2	149	458	5.6	134	89	5	8.9		18	30	37	34	3.74	324	Unremarkable	Yes
19	6.63	4.1	39	15.6	91.81	< 2	137	413	4.5	135	75	5	9.5	4.4	21	26	69		6.4	330		Yes
6	7.03	6.6	25.3	26.6	42.21	< 2	169	415	6	135	95	6	4.5	5.5	11	19	38	35	2.97	356		Yes
2	7.16	9.1	26	7.95	126.55	< 2	175	245	5.3	134	119	7	4.6	7	17	33		18	2.3	328		Yes
22	7.15	10.8	31.5	11	78	< 2	166	288	4.3	135	102	10	5	5	25	24	130	25	2.31			Yes
14	7.1	9.1	29.3	20.3	65.56	< 2	185	378	4.32	131	113	5	9.6	5.3	40	24		29	4.62	325	Unremarkable	Yes
23	7.22	11.9	29.4	6.68	77.4	< 2	168	294	5	131	74	10	4.6	4.4	47	33	56	27	1.3	300		Yes
16					200.93	< 2																No
11	7.27	18.2	40	7.9	77.91	< 2	168	195	3.9	135	69	16	4.8	5	15	20		16	1.5	288		Yes
10	7.18	11.3	31	9.05	103.92	< 2	149	387	4.5	133	83	10	5.3	4.7	22	27	112	24	1.42	315		Yes
4	7.07	7	24.2	8.6	190.49	< 2	188	474	5.4	137	103	6	6.7	6.4	65	32	90	39		.		Yes
5	7.123	8.2	25	16.8	136.26	< 2	171	366	5.4	136	153	5	4.9	7.1	26	31	52	21	2.86	345		Yes
9	7.07	10.9	28.6	15.9	101.16	< 2	181	253	4.4	136	124	7	4.8	5	20	24		31	2.74			Yes
3	7.19	10.9	28.8	13.9	156.36	< 2	168	142	4.6	137	89	9	5.6	2.7	15	22	51	28	1.42	339		Yes
15	7.17	9.6	27.3	13.5	163	< 2	179	201	4.7	136	117	7	5.5	8.5	27	29	94	36	2.52	346		Yes
1	7.21	16.3	40.9	12.3	197	< 2	357	258	4.5	136	90	8	6.8	4.9	14	22		33	2.72	357		Yes
8	7.11	6.4	20.5	14.1	77.53	< 2	16	322	4.2	130	101	5	6.7	3.7	100	39	577	30	1.12			Yes

#	initiation of fom	Dialysis?	many times of di	e to initiation of	NaHCO3?	to NaHCO3 initi	Folate	Intubatetd	Impaired vision	ognitive impairme	Brain death	halmology evalu	isposition from E	Final disposition
17	Within 1 hour	Hemodialysis	1	Within 1 hour	Yes	Within 1 hour	Yes	Yes	No	No	Yes		ICU	Dead
21	Within 1 hour	Hemodialysis	3	Within 5 hours	Yes	Within 1 hour	Yes	Yes	No	No	Yes		ICU	Dead
20	Within 1 hour	ot received dialysis			Yes	Within 1 hour	Yes	Yes	No	No	Yes		Dead	Dead
7	Within 1 hour	Hemodialysis	2	Within 7 hours	Yes	Within 1 hour	Yes	Yes	Yes	Yes	No	Decrease vision	ICU	Home
12	Within 2 hour	Hemodialysis	1	Within 4 hours	Yes	Within 1 hour	Yes	Yes	Yes	Yes	No	Decrease vision	ICU	Home
18	Within 3 hour	Hemodialysis	2	Within 10 hours	Yes	Within 3 hours	Yes	Yes	Yes	Yes	No	Decrease vision	ICU	Home
13	Within 5 hour	Hemodialysis	3	Within 4 hours	Yes	Within 1 hour	Yes	Yes	No	Yes	No		ICU	Home
19		Hemodialysis	1	Within 7 hours	Yes		Yes	Yes	No	Yes	No	DAMA	ICU	DAMA
6	Within 4 hour	Hemodialysis	2	Within 10 hours	Yes	Within 4 hours	Yes	No	Yes	No	No	Decrease vision	ICU	Home
2	Within 3 hour	Hemodialysis	2	Within 8 hours	Yes		No	No	Yes	No	No	Decrease vision	Ward	Home
22	Within 3 hour	Hemodialysis	1	Within 4 hours	Yes	Within 3 hours	Yes	No	Yes	No	No	Decrease vision	Ward	Home
14	Within 1 hour	Hemodialysis	1	Within 4 hours	Yes	Within 1 hour	Yes	Yes	Yes	No	No	Decrease vision	ICU	Home
23	Within 1 hour	Hemodialysis	1	Within 12 hour	Yes	Within 1 hour	Yes	No	No	No	No	Normal exam	Ward	Home
16		Not received dialysis			Yes				No	No	No		Dead	Dead
11	Within 1 hour	ot received dialysis			Yes	Within 1 hour	No	No	No	No	No		Ward	Home
10	Within 1 hour	Hemodialysis	1	Within 12 hours	Yes	Within 1 hour	Yes	No	No	No	No		Ward	Home
4	Within 2 hour	Hemodialysis	2	Within 5 hours	Yes	Within 3 hours	Yes	No	No	No	No		ICU	Home
5		Hemodialysis	1	Within 3 hours	Yes		Yes	No	No	No	No		Ward	Home
9		Hemodialysis	1		Yes	Within 3 hours	Yes	No	No	No	No	DAMA	Ward	DAMA
3	Within 1 hour	Hemodialysis	1	Within 4 hours	Yes	Within 1 hour	Yes	No	No	No	No	Normal exam	Ward	Home
15	Within 2 hour	Hemodialysis	1	Within 4 hours	Yes		Yes	No	No	No	No	Normal exam	Discharge	Home
1		Hemodialysis	2	Within 4 hours	Yes		Yes	No	No	No	No	Normal exam	Ward	Home
8	Within 1 hour	Hemodialysis	1	Within 8 hours	Yes	Within 2 hours	No	Yes	No	No	No	Normal exam	ICU	Home

Clinial data for sample patient.
